# Familial juvenile polyposis syndrome with a de novo germline missense variant in *BMPR1A* gene: a case report

**DOI:** 10.1186/s12881-020-01135-6

**Published:** 2020-10-08

**Authors:** Qing Liu, Mengling Liu, Tianshu Liu, Yiyi Yu

**Affiliations:** Department of Medical Oncology, Zhongshan Hospital, Fudan University, 180 Fenglin Road, Shanghai, 200032 China

**Keywords:** Juvenile polyposis syndrome, *BMPR1A* gene, De novo germline variant, Missense variant

## Abstract

**Background:**

Juvenile polyposis syndrome (JPS) is a rare autosomal dominant hereditary disorder characterized by the development of multiple distinct juvenile polyps in the gastrointestinal tract with an increased risk of colorectal cancer. Germline mutations in two genes, *SMAD4* and *BMPR1A*, have been identified to cause JPS.

**Case presentation:**

Here, we report a germline heterozygous missense variant (c.299G > A) in exon 3 *BMPR1A* gene in a family with juvenile polyposis. This variant was absent from the population database, and concluded as de novo compared with the parental sequencing. Further sequencing of the proband’s children confirmed the segregation of this variant with the disease, while the variant was also predicted to have damaging effect based on online prediction tools. Therefore, this variant was classified as likely pathogenic according to the American College of Medical Genetics and Genomics (ACMG) guidelines.

**Conclusions:**

Germline genetic testing revealed a de novo germline missense variant in *BMPR1A* gene in a family with juvenile polyposis. Identification of the pathogenic variant facilitates the cancer risk management of at-risk family members, and endoscopic surveillance is recommended for mutation carriers.

## Background

Juvenile polyposis syndrome (JPS) is a rare autosomal dominant hereditary disorder characterized by the development of multiple distinct juvenile polyps in the gastrointestinal tract with an increased risk of colorectal cancer [1, 2]. Clinically, JPS is defined by the presence of more than five juvenile polyps in the colorectum, and/or juvenile polyps outside the colon, and/or any number of juvenile polyps with a family history of juvenile polyposis [3]. Histologically, these polyps are characterized by an abundance of edematous lamina propria with mucin-filled cystic dilations and inflammatory infiltrate [4]. Germline mutations in two genes, *SMAD4* and *BMPR1A*, have been identified to cause JPS [5]. Both genes are members of the transforming growth factor beta (TGF-β) superfamily, and pathogenic mutations in the coding region of each gene have been found in ~ 20% of JPS patients, respectively [6]. Here we report a de novo germline missense variant in *BMPR1A* gene in a family with juvenile polyposis.

## Case presentation

**Fig. 1:**
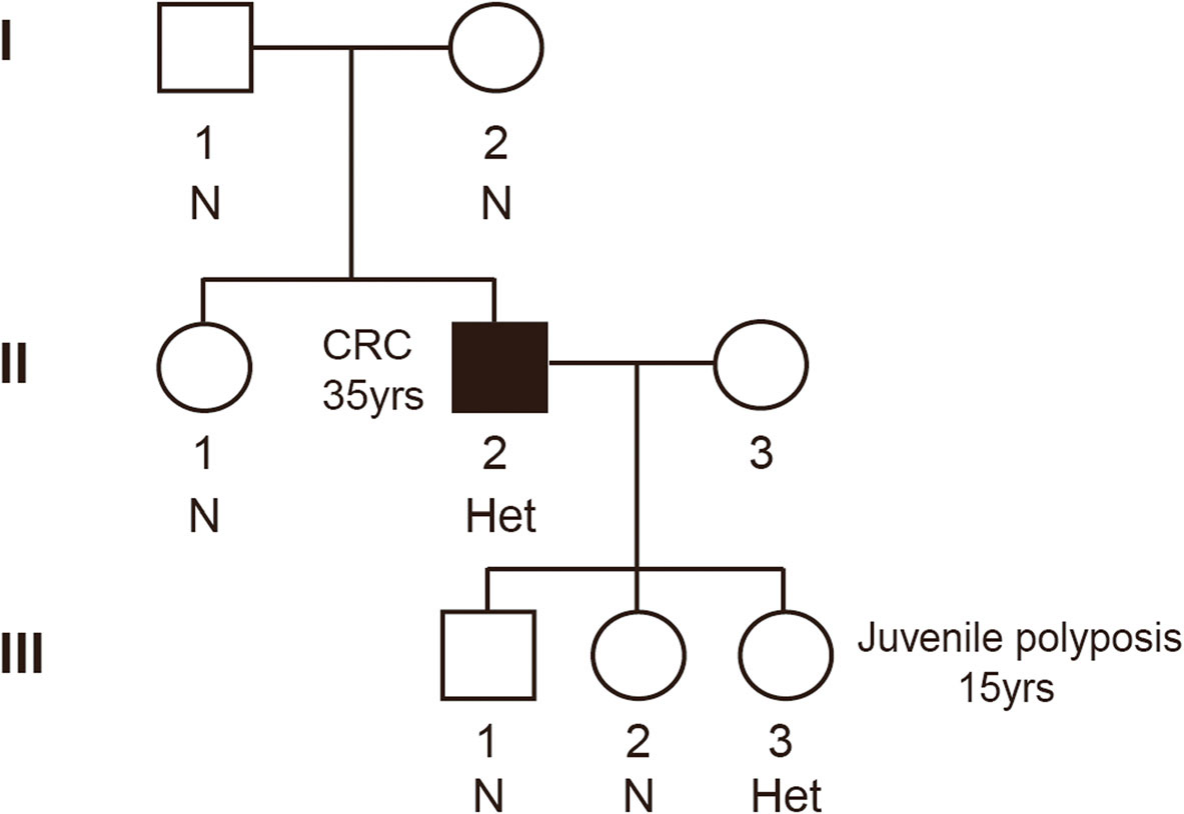
The pedigree of the family investigated in this study, with the proband indicated by the black shading. Age of onset is noted beside the family member. The status of *BMPR1A* variant is listed beneath each family member. CRC, colorectal cancer. N, none. Het, heterozygous

The 35-year-old male proband was first presented with rectal bleeding for 2 months in September 2015 (Fig. 1). Colonoscopy was then conducted and revealed dozens of pedunculated polyps of different sizes (range of 5–30 mm), distributed along the entire length of the colon. The histological analysis showed juvenile and adenomatous polyp with low-grade dysplasia. The proband received a right hemicolectomy in January 2016 for a T3N0M0 moderately differentiated adenomatous carcinoma of the transverse colon but developed liver and lung metastasis in 2018.

The patient’s parents (I-1, I-2) and his sister (II-1) were healthy without any symptoms when the proband was diagnosed with colorectal cancer (CRC). His three children (III-1, III-2 and III-3) had colonoscopy in 2018 (at the age of 12, 18 and 15 respectively), one of which (III-3) was found to have 3 polyps. Endoscopic mucosal resection was then performed to fully remove these polyps and histology was consistent with juvenile polyp.

### Germline genetic testing

**Fig. 2:**
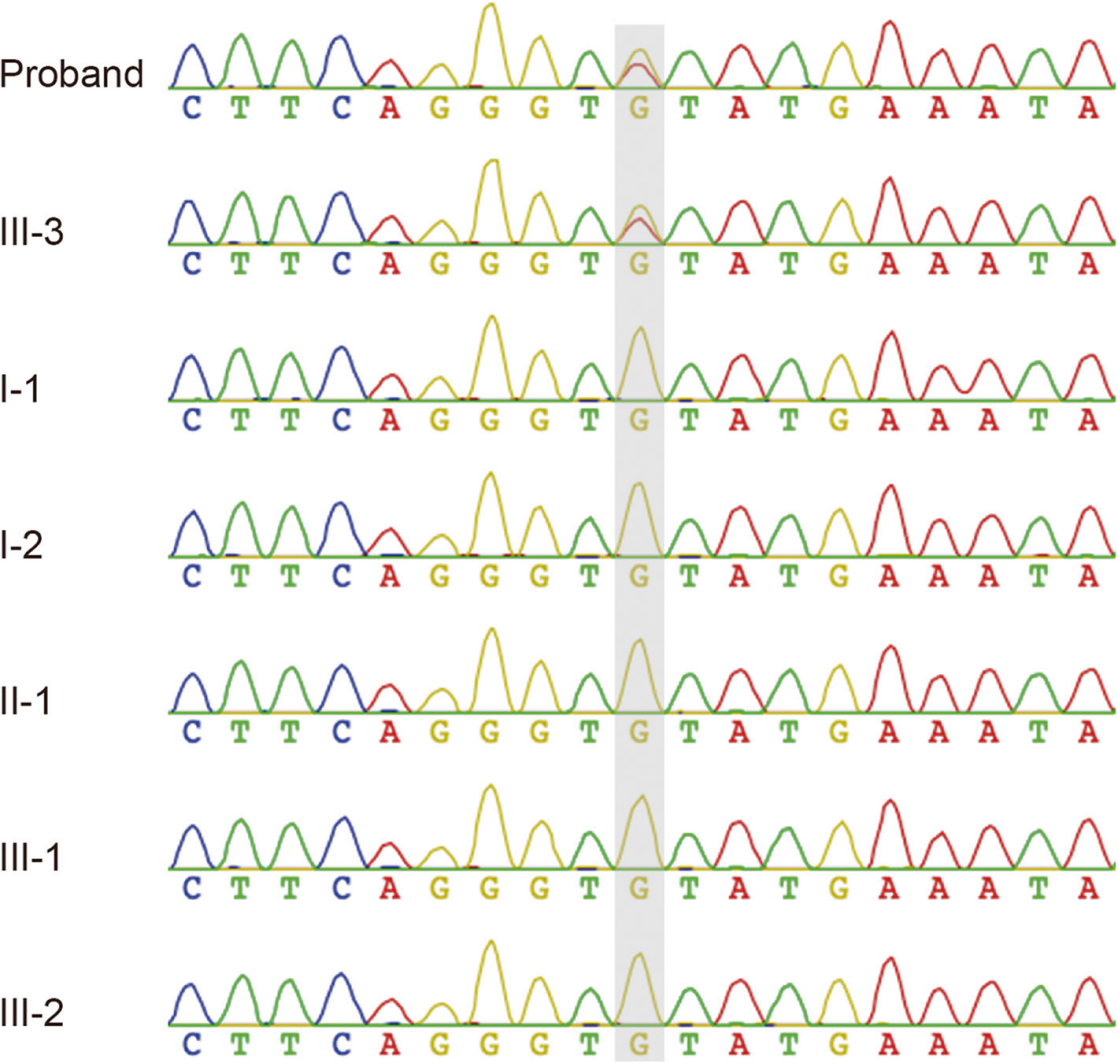
Missense variant (c.299G > A) in the *BMPR1A* gene confirmed by sanger sequencing of the family

Given the clinicopathological findings and the family history, the diagnosis was familial juvenile polyposis. Subsequently, germline genetic testing via a multigene panel (66 genes), which included genes associated with hereditary tumors such as *APC*, *BMPR1A*, *BRCA1*, *BRCA2*, MMR genes, *MUTYH*, *PTEN*, *SMAD4*, *STK11*, *POLD1* and *POLE*, was performed on the proband and his family members. Finally, an unreported heterozygous c.299G > A (p.Cys100Tyr) missense mutation in exon 3 of the *BMPR1A* gene (NM_004329) was identified. The variant was further confirmed by Sanger sequencing (Fig. 2), and concluded as de novo compared with the parental sequencing. The c.299G > A (p.Cys100Tyr) variant was absent from the dbSNP, the 1000G, ESP and ExAC databases. Furthermore, this variant was predicted as damaging according to online prediction tools including SIFT, Polyphen2 and Mutation Taster. Moreover, the proband’s two children (III-1, III-2) who had negative findings from colonoscopy did not carry this variant. Only the individual III-3 who presented with polyps had this variant, indicating the co-segregation of the variant in this family (Fig. 1).

According to the latest American College of Medical Genetics and Genomics (ACMG) guidelines for the interpretation of sequence results, this variant in BMPR1A gene fulfills PS2, PM2, PP1 and PP3 and is therefore regarded as likely pathogenic [7].

## Discussion and conclusions

BMPR1A is a serine-threonine kinase receptor, with a cysteine-rich extracellular region, an intracellular glycine-serine-rich domain, and an intracellular kinase domain [8]. It is involved in the TGF-β signaling pathway which is an important regulator of various cellular processes, including proliferation, differentiation, migration and death [9]. Several different types of mutations of the *BMPR1A* gene have been identified in JPS patients, including large deletions, missense and nonsense substitutions, and small indels that result in frameshift mutations [1, 6, 10]. However, the functional consequences of missense variants are not always as obvious. The substitution of one amino acid for another may or may not have deleterious effect on the structural properties with the corresponding proteins. Hence, such variants are regarded as “variants of uncertain significance” (VUS).

Several studies have characterized the feature of missense variants in *BMPR1A* gene functionally in vitro. Using confocal microscopy and luciferase assays, Howe et al. [11] found that missense variants in *BMPR1A* could have damaging impact on the localization of the protein to cell membrane instead of reducing protein levels. Kotzsch et al. [12] showed that extracellular domain variants could inactivate BMP-2 signaling by depriving their folding ability compared with wild-type protein. Yet the pathogenicity of these variants in vivo is still inconclusive. A further study is needed, i.e. functional study to prove that this new de novo missense variant in exon 3 *BMPR1A* gene in this study is pathogenic variant. Missense variants of *BMPR1A* gene identified in JPS patients reported in PubMed from 2000 to 2019, including the present case, are summarized in Table 1, with the pathogenicity evaluated according to ACMG guidelines [6, 10, 13–25]. Most of the previous reported missense variants are classified as VUS except for c.1328G > A, another de novo missense variant listed as likely pathogenic [24].

**Table 1:** Missense Variants of *BMPR1A* gene identified in JPS patients

Nucleotide variant	dbSNP number	Protein change	ACMG guideline [7]	Pathogenicity	Ref
c.1A > C	rs786203157	p.Met1Leu	PM2 PP3	VUS	[13]
c.4C > A	rs11528010	p.Pro2Thr	BA1	benign	[10]
c.170C > G	rs1057517610	p.Pro57Arg	PM2 PP3	VUS	[6, 13, 14]
c.184 T > G	/	p.Tyr62Asp	PM2 PP3	VUS	[6, 14, 15]
c.233C > T	rs1064793490	p.Thr78Ile	PM2 PP1 PP3	VUS	[6, 13, 16]
c.238G > A	/	p.Gly80Arg	PM2	VUS	[17]
c.245G > A	/	p.Cys82Tyr	PM2 PP3	VUS	[6, 13, 15]
c.299G > A	/	p.Cys100Tyr	PS2 PM2 PP1 PP3	likely pathogenic	this study
c.355C > T	rs587782494	p.Arg119Cys	PM2	VUS	[17]
c.359G > C	/	p.Arg120Pro	PM2 PP3	VUS	[18]
c.370 T > C	rs199476087	p.Cys124Arg	PM2	VUS	[19]
c.373 T > G	rs1131691180	p.Cys125Gly	PM2 PP3	VUS	[18]
c.385 T > A	/	p.Leu129Ile	PM2 BP4	VUS	[18]
c.388 T > C	rs1131691168	p.Cys130Arg	PM2 PP3	VUS	[17, 20]
c.524G > A	rs370091063	p.Cys175Tyr	PP3 BP6	VUS	[21]
c.761G > A	rs766908700	p.Arg254His	PP3 BS2	VUS	[13]
c.872 T > C	/	p.Phe291Ser	PM2 PP3	VUS	[18]
c.955 T > C	/	p.Leu332Pro	PM2 PP3	VUS	[22]
c.1013C > A	rs199476086	p.Ala338Asp	PM2 PP3	VUS	[6, 15, 19]
c.1058A > G	rs1405441693	p.Gln353Arg	PM2 PP3	VUS	[22]
c.1127G > A	rs199476088	p.Cys376Tyr	PM2 PP3	VUS	[19]
c.1229C > T	/	p.Pro410Leu	PM2	VUS	[17]
c.1231G > A	rs786202611	p.Glu411Lys	PM2 PP3	VUS	[23]
c.1242G > A	rs140592056	p.Glu415Lys	PP3	VUS	[18]
c.1327C > T	rs35619497	p.Arg443Lys	PP3 PP5 BP6	VUS	[6, 13, 15, 18]
c.1328G > A	rs876659155	p.Arg443His	PS2 PM2 PP3	likely pathogenic	[24]
c.1409 T > C	rs199476089	p.Met470Thr	PM2 PP3	VUS	[25]
c.1433G > A	rs113849804	p.Arg478His	PP3	VUS	[22]
c.1438C > T	rs876658515	p.Arg480Trp	PM2 PM5 PP3	VUS	[10]

Here, we identified a germline missense variant in *BMPR1A* gene in a family with juvenile polyposis. This variant is classified as likely pathogenic variant based on multiple lines of evidence. First, absence of the c.299G > A (p.Cys100Tyr) variant in both parents validated its de novo status. Further sequencing of the proband’s children confirmed the segregation of this variant with the disease. Second, no missense variant has been reported in the population databases at this position. Third, the p.Cys100Tyr is located within the cysteine-rich domain, a highly-conserved ectodomain of the TGF-β receptor family, which is very likely to result in conformational alterations (suppl Fig. 1) [26]. In silico analysis using multiple computational tools also shows damaging effect of this variant.

For patients with JPS, endoscopic surveillance should be performed yearly until the patient is deemed to be polyp free [27]. Therefore, genetic testing for at-risk family members is an important procedure in the management. In this case, the proband’s daughter (III-3) who carry the missense variant should receive high-risk surveillance to prevent the development of cancer, while his other two children (noncarriers) may no longer require close endoscopic screening.

In summary, we report a de novo germline heterozygous missense variant in exon 3 *BMPR1A* gene in a family with juvenile polyposis. Identification of the pathogenic variant facilitates the cancer risk management of at-risk family members, and endoscopic surveillance is recommended for mutation carriers.

## Supplementary information

**Additional file 1 **: **Suppl Fig. 1** The upper rectangle represents the different domains of the *BMPR1A* gene. The lower panel shows analysis of evolutionary conserved amino acids in human BMPR1A protein predicted by ConSurf.

## Data Availability

The raw datasets generated and/or analyzed during the current study are not publicly available in order to protect participant confidentiality, but are available from the corresponding author on reasonable request.

## Abbreviations

JPS: Juvenile polyposis syndrome
ACMG: American College of Medical Genetics and Genomics;
TGF-β: Transforming growth factor beta
CRC: Colorectal cancer
VUS: Variants of uncertain significance
