# Supplementary figures and images for: Familial juvenile polyposis syndrome with a de novo germline missense variant in BMPR1A gene: a case report
Source: BMC Med Genet. 2020 Oct 8;21:196. doi: 10.1186/s12881-020-01135-6 (PMC7545562; doi:10.1186/s12881-020-01135-6)

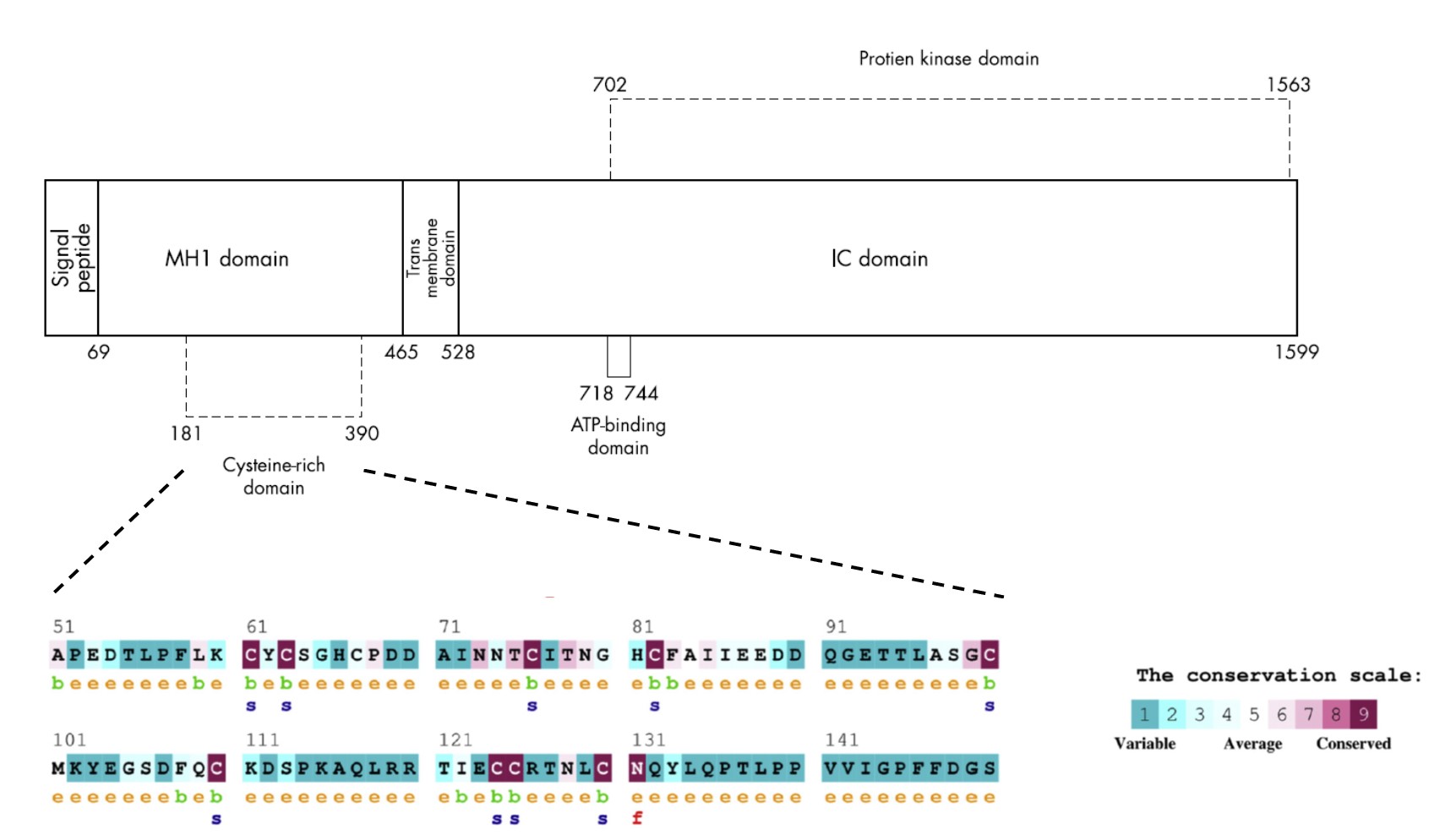

Supplement: Supplementary file 1 — Additional file 1 : Suppl Fig. 1 The upper rectangle represents the different domains of the BMPR1A gene. The lower panel shows analysis of evolutionary conserved amino acids in human BMPR1A protein predicted by ConSurf. [file 12881_2020_1135_MOESM1_ESM.jpg]
